# PregTox: A Resource of Knowledge about Drug Fetal Toxicity

**DOI:** 10.1155/2022/4284146

**Published:** 2022-04-16

**Authors:** Qingqing Chen, Yu Gan, Kejian Wang, Qing Li

**Affiliations:** ^1^Department of Obstetrics and Gynecology, Key Laboratory for Major Obstetric Diseases of Guangdong Province, The Third Affiliated Hospital of Guangzhou Medical University, Duobao Road 63, Guangzhou, 510150, Guangdong, China; ^2^The Third Affiliated Hospital of Shandong First Medical University (Affiliated Hospital of Shandong Academy of Medical Sciences), Jinan, Shandong, China; ^3^Gastroenterology Institute and Clinical Center of Shandong First Medical University, Jinan, Shandong, China; ^4^Innovative Institute of Chinese Medicine and Pharmacy, Shandong University of Traditional Chinese Medicine, Jinan, Shandong, China

## Abstract

**Background:**

It is of vital importance to determine the safety of drugs. Pregnant women, as a special group, need to evaluate the effects of drugs on pregnant women as well as the fetus. The use of drugs during pregnancy may be subject to fetal toxicity, thus affecting the development of the fetus or even leading to stillbirth. The U.S. Food and Drug Administration (FDA) issued a toxicity rating for drugs used during pregnancy in 1979. These toxicity ratings are denoted by the letters A, B, C, D, and X. However, the query of drug pregnancy category has yet to be well established as electronic service.

**Results:**

Here, we presented PregTox, a publicly accessible resource for pregnancy category information of 1114 drugs. The PregTox database also included chemical structures, important physico-chemical properties, protein targets, and relevant signaling pathways. An advantage of the database is multiple search options which allow systematic analyses. In a case study, we demonstrated that a set of chemical descriptors could effectively discriminate high-risk drugs from others (area under ROC curve reached 0.81).

**Conclusions:**

PregTox can serve as a unique drug safety data source for drug development and pharmacological research.

## 1. Introduction

The safety of a drug is as important as its efficacy [1]. Pharmaceutical companies consistently invest numerous material, financial, and human resources into clinical trials to discover the possible safety risks of drugs [2]. Pregnant women, as a special group of people, are more rigorous in clinical trials, and some drugs cannot be tested due to unknown safety risks [3, 4]. In many cases, pregnant women need to take medication for a combination of medical conditions. As drug toxicity may harm not only the mothers but also the fetuses, by affecting the intrauterine growth and inducing malformation or even stillbirth. Nevertheless, the vast majority of drugs have yet to be clarified for fetal toxicity risks due to limited pharmacological and clinical evidence.

Thalidomide was initially marketed as a treatment for hyperemesis gravidarum and received favorable reviews due to its low hepatotoxicity. But in subsequent years, there was a succession of malformed fetus who had no arms or legs and had hands and feet attached directly to their bodies. The drug is classified as class X in pregnancy, which means thalidomide is not allowed during pregnancy [5–7]. Thalidomide also has some other side effects, such as heart and urinary tract abnormalities, blindness, and deafness [8]. The researchers proposed several possible reasons for the formation of fetal deformities caused by thalidomide, such as DNA mutagenesis, disturbance on chondrogenesis, or inhibition of cell adhesion [9–11]. Thalidomide incident brought heavy disaster to the society and the family. Higher and more stringent requirements were put forward for drug development and use during pregnancy [12].

In many cases, medication use during pregnancy is unavoidable. It is estimated that 1% to 3% of the newborns are subject to various birth defects, among which 2–3% are related to drugs used in the course of pregnancy [13]. A study on 1626 pregnant women suggested that 56% of the participants used prescription drugs [14]. Another study on 205 pregnant and recently delivered women reported an average of over 3 prescription drugs received during pregnancy [15]. Therefore, it is of great importance for clinicians to know the accurate fetal toxicity information of drugs, so as to help patients make informed decisions. And the knowledge on fetal toxicity of marketed drugs can help pharmaceutical industry rule out the risky compounds in future research and development efforts [16, 17].

In order to warn the risk of drug-induced fetal injury, the U.S. FDA established category labels in 1979 [18], which consist of five ranks (i.e., A, B, C, D, and X) standing for escalating risk levels. This classification system has been widely accepted in the United States and around the world. Category A indicates that adequate and well-controlled studies in pregnancy women showed no adverse effects on fetus. Category B indicates that animal reproduction studies demonstrated no risk to the fetus, while no adequate well-controlled studies in pregnant women were conducted. Category C indicates that no adequate and well-controlled studies in pregnancy women showed risk, but animal studies indicated adverse effect on fetus. Category D indicates evidence of human fetal risk based on adverse reaction data from investigational and marketing experience or studies in humans. Potential benefits may warrant use of the drugs of categories C and D during pregnancy despite potential risks. Category X indicates that controlled studies in animals or humans have demonstrated fetal abnormalities; thus, the risks clearly outweighed potential benefits. Several public resources contain information relevant to drug toxicity (e.g., websites from regulatory agencies, World Health Organization's consolidated list for withdrawn drugs, and scientific literature). Even so, in most circumstances, the information is hidden in regulatory documents and not easily accessible, thus impeding comprehensive analyses based on a complete list of risky drugs.

With the development of information technology, a plenty of databases have been built in Asia and all over the world to facilitate biomedical research. For instance, DockCoV2 is a database of compounds against SARS-CoV-2, which aims at speeding up the discovery of potential drugs [19]. Similarly, DDInter is an online drug–drug interaction database towards improving clinical decision-making and patient safety [20]. And ADReCS is an ontology database for the purpose of standardization and hierarchical classification of adverse drug reaction terms [21]. These examples suggested that a bioinformatics database with comprehensive data and powerful visualization tools could provide a highly useful research platform for clinicians and drug developers.

Based on the current uncertainty about fetal toxicity of some drugs during pregnancy and to provide a user-friendly access to drug label information and facilitate data-driven drug safety research, here, we present PregTox—a resource of knowledge about drug fetal toxicity. A total of 1114 drugs were collected with category labels, chemical characteristics, and clinical information. Furthermore, PregTox provides multiple search options to systematically analyze molecules of interest for drug development and toxicity prediction.

## 2. Methods

### 2.1. Data Collection

**Figure 1:**
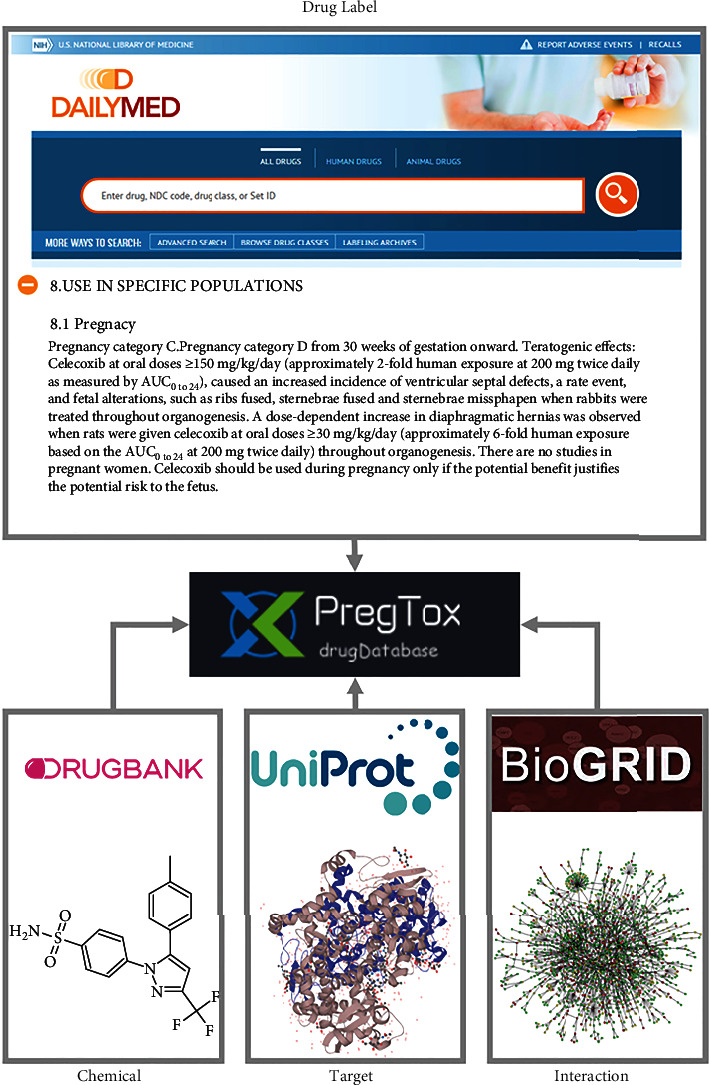
The derived informational resources of PregTox database.

The pregnancy category information was extracted from the DailyMed database (https://dailymed.nlm.nih.gov/dailymed) and manually curated by two independent researchers (inconsistencies were resolved by a third researcher). We ultimately defined 1114 drugs that had pregnancy categories. For drugs corresponding to two or more categories (e.g., rabeprazole corresponds to both categories “B” and “C” from different packagers), the category indicating the higher risk was selected. Other basic drug information was extracted from DrugBank [22], UniProt [23], and BioGRID [24] databases (Figure 1).

### 2.2. Database Construction

PregTox is based on a nonrelational MongoDB database with high performance (https://www.mongodb.com/). All data concerning PregTox is stored on the MongoDB database, and PregTox is hosted on a Linux virtual server as a Go web application which was compiled into a binary executable file, accessible at http://pregtox.gzhmu.edu.cn. Although we make every effort to solve many cross-browser compatibility issues, limited by the poor support ability for the new feature of a low version browser, we highly suggest using the latest version of Mozilla Firefox, Google Chrome, or Microsoft Edge browser with JavaScript option enabled for normal visual presentation.

### 2.3. Statistical Analyses

The molecular descriptors of drugs were generated by ChemDes online server [25]. QSAR data were analyzed in the R software (version 4.0.3). The following functions or packages were used in our analyses: Principal component analysis was carried out with the “PCA” function in the “FactoMine” R package and the “fviz_pca_ind” function in “Factoextra” package. Analysis of similarities (ANOSIM) was performed with the “vegan” package. Leave-one-out cross-validation of *k*-nearest neighborhood model was carried out with the “kknn” and “class” package. And the “ROCR” package was used to calculate the area under ROC curve.

## 3. Results

### 3.1. Data Summary

**Figure 2:**
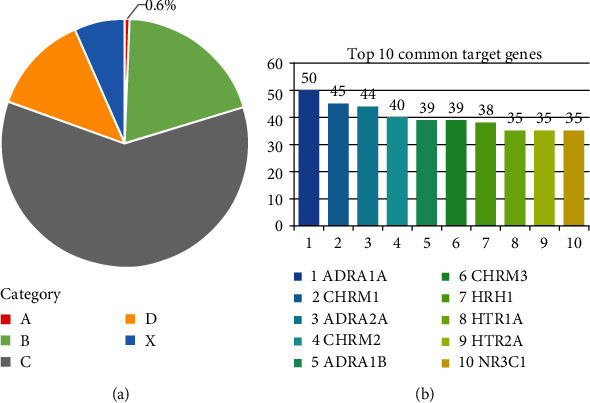
Statistics of PregTox contents: (a) distributions of drug categories; (b) the top 10 common drug target genes.

The current version of PregTox encompasses various knowledge of 1114 drugs using in pregnancy. The risk category information was curated from FDA drug labels, with category C accounting for the largest proportion of drugs (60.2%), followed by category B (19.7%), category D (12.9%), category X (6.6%), and category A (less than 1%, Figure 2(a)). The top common target genes are mostly neurotransmitter receptors (e.g., adrenoceptors, cholinergic receptors, and 5-HT receptors, as shown in Figure 2(b)). The data thus suggested that drugs with uncertain risks constitute the bulk of approved drugs, and the well-known risky and safe drugs are both interacted with a variety of target proteins.

### 3.2. User Interface

**Figure 3:**
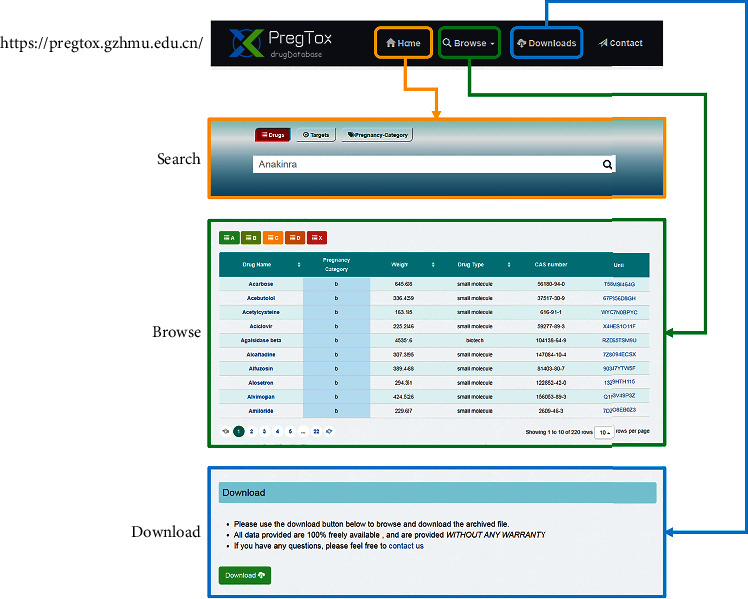
Overview of PregTox user interface.

The PregTox website is characterized by a user-friendly interface for exploring and visualizing drug information. The homepage has links to “Search,” “Browse,” “Downloads,” and “Contact” functions (Figure 3). The “Browse” page provides the entry to explore the datasets by different drugs, therapeutic targets, or risk categories. When browsing a specific drug, all related information is shown in a single page, such as chemical properties, drug indications, and drug targets, along with pregnancy category information. Particularly, the protein-protein interactions (PPI) were retrieved from the BioGRID database, which can help the analysis and visualization of drug-gene network. Furthermore, there are external links pointing to the sources (e.g., DrugBank, UniProt, and BioGRID) of certain information, so as to facilitate in-depth exploration. The complete list of drugs can be obtained by clicking on the “Downloads” link in the homepage, which enables data mining and training of prediction models. In case of any inquiries or technical problems, the email address of administrators can be found in the “Contact” page.

### 3.3. Application Case: Naive Machine Learning on Fetal Toxicity Risks

**Figure 4:**
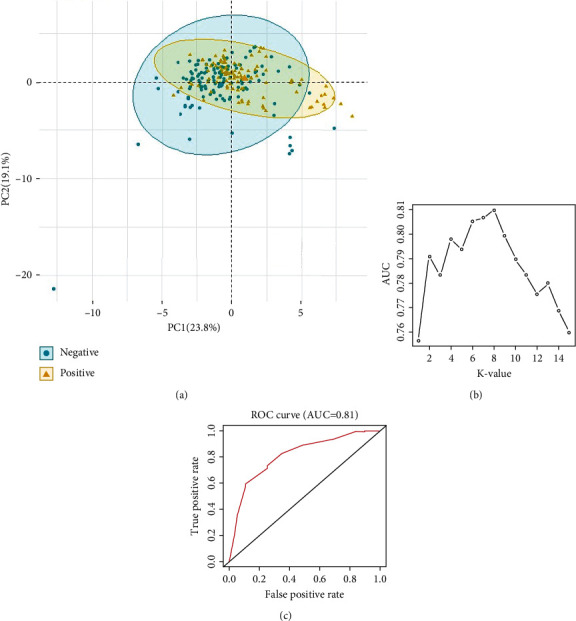
Application of PregTox data. (a) PCA plot demonstrated a significant separation between positive and negative compounds. (b, c) After selecting the optimal *k* number, the KNN model achieved an AUC of 0.81.

To illustrate the utility of PregTox, we performed a pilot analysis on the 2D molecular descriptors of compounds in the data inventory. Using categories A and B as positive data set while categories D and X as negative set, we analyzed the association between drug chemical structure and fetal toxicity. We first preformed the principal component analysis (PCA) to summarize patterns of multivariate variation between drugs. The results showed a significant separation between the positive and negative datasets (Figure 4(a), ANOSIM *P* = 0.002). Additional analysis was carried out by training anaïvek-nearest neighborhood (KNN) classification model with the chemical features. With *k* = 8 that gave the best performance (Figure 4(b)), leave-one-out cross-validation suggested that area under the ROC curve (AUC) reached 0.81 (Figure 4(c)). This application case indicated that data stored in PregTox could serve as a unique resource to support fetal safety assessment on candidate drugs under development.

## 4. Discussion

PregTox is a rich resource of knowledge about fetal toxicity of drugs. Due to a number of drugs reported for previously unknown fetal toxicity, we will continually update the database to ensure coverage and accuracy of information. The PregTox contains not only FDA-established category labels of fetal toxicity, but also chemical features, defined daily dose and drug-target information. Such information can expand the usefulness of PregTox. For instance, the illustrated application case suggested that connecting risk category labels and drug chemical structures could help build up the QSAR models to predict the risk of fetal injury for new drugs.

Traditional methods for drug safety evaluation are generally cost-ineffective, time-consuming, and labor-intensive, so various computational approaches have been developed to predict drug toxicity. In recently years, technological advancements motivated detection of drug safety risks based on deep learning methods [26–29]. However, well-labeled data for model training remain scarce resources. Even though a variety of pharmacopeias databases (e.g., DrugBank [22], TTD [30], VARIDT [31], INTEDE [32], and ClinicalTrials.gov) have been developed to provide data sources for bioinformatics analysis, the lack of authoritative, comprehensive, and structured data in fetal toxicity hinders professionals from conveniently analyzed the chemical and biological features of potentially risky drugs. These prior works showed that machine learning techniques can be harnessed with a good description of chemical descriptors to more efficiently process large amounts of data [33, 34]. We believe that the data in PregTox can provide specific information on drug fetal toxicity, which enabled naïve machine learning models to achieve a desired level of performance. Therefore, it is reasonable to expect broader applications of the PregTox database in deep learning. Moreover, the rationale of PregTox could shed lights on new frontiers in various types of drug toxicity. A well-structured data source will also facilitate the intensive data mining on cardiotoxicity, drug-induced liver injury, and other risks.

## 5. Conclusions

In this context, the knowledge presented in PregTox will facilitate systematic analysis on chemical and biological characteristics of drugs with fetal toxic effects. Also, PregTox can serve as a data source for safety assessment during drug development and scientific research in mechanisms of drug-induced fetal toxicity.

## Data Availability

The pregnancy category information of drugs data used to support the findings of this study are included within the article. Also, it can be found on PregTox database (https://pregtox.gzhmu.edu.cn).

## Abbreviations

FDA: Food and Drug Administration.
